# The Transition to a Coresidential Partnership: Who Moves and Who Has the Partner Move In?

**DOI:** 10.1007/s11113-021-09665-4

**Published:** 2021-07-11

**Authors:** Sandra Krapf, Clara H. Mulder, Michael Wagner

**Affiliations:** 1 Mannheim Centre for European Social Research, University of Mannheim, Mannheim, Germany; 2 Population Research Centre, Faculty of Spatial Sciences, University of Groningen, Groningen, The Netherlands; 3 Institute of Sociology and Social Psychology, University of Cologne, Cologne, Germany

**Keywords:** Coresidence, Union formation, Relocation, Migration

## Abstract

Moving into a joint household is an important step in the process of union formation. While a growing body of literature investigates differences between those couples who start coresidence and those who do not, we know little about the likelihood of moving upon the start of coresidence. The aim of this paper is to investigate how individual and couple-level characteristics are associated with moving, or having a partner move in, at the start of coresidence. We use data from 10 waves of the German Family Panel pairfam for those who started coresidence (*n* = 983) and estimate logistic regression models of moving versus having a partner move in. The respondents in the sample are quite young with a mean age of 27. For long-distance relationships, those with a higher level of education than their partner and women who were living in close proximity to their parents were less likely to move. In short-distance relationships, respondents living in the parental home or in crowded housing were more likely to move than those living in uncrowded housing. In contrast with previous research, we did not find that women were more likely to move than men. Our results highlight that factors like educational resources, housing demands, and local family ties have differential effects on moving decisions at the start of coresidence depending on the distance moved.

## Introduction

The transition to coresidence is a crucial event in the process of partnership and family formation. Although an increasing share of couples in many western European countries have a child before they marry, the vast majority of couples live in a joint household before a child is born (Kiernan, 2001; Mikolai, 2017). A growing number of studies have investigated which individual and couple characteristics are related to the probability of starting a joint household versus remaining in a living-apart-together relationship or separating (Krapf, 2018; Régnier-Loilier, 2016; Sassler et al., 2016; Wagner et al., 2019). However, hardly any studies have investigated the question of who moves at the start of coresidence and who has their partner move in (Brandén & Haandrikman, 2019, for an exception). There are some studies addressing moving upon marriage (e.g.Fan & Huang, 1998; Mulder & Wagner, 1993), but these studies do not take into account the characteristics of both partners.

The issue of who moves upon coresidence is important because the partner who moves might be in a less advantaged position than the partner who does not. With regard to long-distance moves, previous research shows that persons who moved for reasons that were unrelated to work, including those who moved in order to live with their partner, had decreasing levels of job satisfaction after the move (Switek, 2016). Moreover, movers might experience difficulties in establishing strong social ties at the new place and have lower levels of well-being (Oishi, 2010). Only with regard to housing satisfaction does relocation seem to have a lasting positive effect (Wolbring, 2017). However, the potential increase in housing satisfaction for the moving partner is likely to be reversed in case of separation. Previous research shows that, in case of union dissolution, an ex-partner who moved in with the other partner at the start of coresidence has an increased probability of leaving the joint home (Mulder & Wagner, 2012). Because moving after separation is often related to downward mobility on the housing ladder (Feijten, 2005; Lersch & Vidal, 2014; Mikolai & Kulu, 2018), the person who moved to the partner’s home at the start of coresidence is more likely to bear negative consequences in the event of separation than the other partner.

The aim of this study is to investigate the likelihood of moving upon the start of coresidence by using the German family panel study pairfam. With a focus on partnership processes of young couples, pairfam is among the very few survey datasets that collects data about partnerships before coresidence starts. One drawback of the data is that we only have information about the moves of the main respondent (the so-called anchor), but not the moves of their partners. Ideally, we would like to distinguish between (a) couples in which the anchor stays and has the partner move in, (b) couples in which the anchor person moves into the partner’s household and (c) those in which both partners move to a new place. Starting from the assumption that moving is generally more costly than having the partner move in, couples of type (b) and (c) differ: in case (b), only the anchor person bears the cost of moving, while in case (c) both partners do. Because of this limitation, we group together types (b) and (c), and focus on a binary variable distinguishing couples in which the anchor person moves from those in which their partner moves in. Our central research question is: Which characteristics are associated with a relocation of the anchor person at the start of coresidence, compared with having the partner move in? Although the answer to this question does not paint a complete picture of who moves at the start of coresidence—one partner, the other partner, or both—it still provides important information on whether one of the partners—the anchor respondent—avoids the cost of moving. The previous study by Brandén and Haandrikman (2019) employs the same type of dependent variable.

Just like the previous study by Brandén and Haandrikman (2019), our study is different from older work on marriage migration in that it considers the characteristics of both partners in a newly formed couple. Compared with Brandén and Haandrikman (2019), a major contribution of our study is that our analysis includes all cohabiting couples, not only those that married or had a child. Furthermore, we analyze a comparably young sample of persons with a mean age of 27. Younger persons in relationships that do not necessarily lead to marriage or parenthood may take different factors into account when deciding who moves at the start of coresidence. First, the initial household and housing situation varies depending on age. Younger persons are more likely to live with their parents (Konietzka & Tatjes, 2014) or in apartments that are too small to start a joint household. Second, the moving decisions of younger couples might follow more gender-egalitarian patterns than those of older couples. The family migration literature shows that women experience moves that are negative for their individual benefit more often than men because they are expected to do so (Cooke, 2008b). Younger adults that are in a life stage before family formation tend to have less traditional gender role attitudes (Scarborough et al., 2019) and therefore, they might differ in their moving decisions from older adults.

For our statistical analyses, we apply logistic regression models to ten waves (2008/2009–2017/2018) of the German Family Panel pairfam. The sample consists of persons who have started coresidence with their partner since the last interview.

## Theoretical Considerations

The focus of this study is on couples who have experienced the transition from a living-apart-together (LAT) relationship to a coresidential partnership. LAT relationships refer to unmarried partnerships in which the partners have a stable intimate relationship but do not live together (Duncan & Phillips, 2010; Mortelmans et al., 2015). The majority of LAT relationships can be seen as a stage on the way to making a stronger commitment and establishing a joint household (Liefbroer et al., 2015). Our analysis focuses on those persons who have made this commitment and have started coresidence. Our research interest is to identify the determinants of moving versus having the partner move in at the start of coresidence. Compared with household moves, this is a special case as the partners do not live in a joint household before the move. Still, we expect that the moving decision is made by both partners jointly because the move has consequences for both partners’ living situation. We employ theoretical arguments from the family migration and relocation literature.

From the perspective of microeconomics, couples relocate if the expected gains of relocation on the couple level exceeds the cost (Mincer, 1978). This is the case for both short- and long-distance moves. However, the costs and benefits of moving differ depending on the distance moved. Families making short distance moves often choose to do so because their current dwelling does not fulfill their (future) housing needs (Rossi, 1955). Thus, the likelihood of moving increases with a change of household composition. Indeed, many empirical studies show that moves are associated with life course events, such as childbirth, marriage or divorce (Clark & Huang, 2004; Cooke et al., 2016; Dommermuth & Klüsener, 2019; Kulu & Steele, 2013; Mulder & Wagner, 1993). Starting coresidence also changes the household composition and practical considerations might play a role when deciding who moves. For example, the dwelling of a particular partner might be too small for a joint household. Therefore, we expect that persons who live in a small dwelling are more likely to move at the start of coresidence compared to persons with a larger dwelling (Hypothesis 1a). If a person lives in the parental home before coresidence starts, the person may prefer to move out of the parental home even if crowding is not a problem because the couple wants to live independently. Persons who live in the parental home are therefore expected to have a higher probability of moving than those living in their own household (H1b).

While these practical reasons are associated with the benefits of moving, moves also incur costs. First, there are the actual moving costs, including the opportunity cost of spending time on arranging the move as well as the direct cost of transporting one’s belongings. Second, there may be other costs related to the move, such as the costs of furnishing and home improvement. Third, long-distance moves create considerable indirect costs because they affect the working and social life of the person who moves (Niedomysl & Fransson, 2014). With reference to social life, a long-distance move entails living farther away from a person’s social network of family and/or friends (DaVanzo, 1981; Mulder & Malmberg, 2014). Although communication technologies have developed rapidly in the last few decades, informal support and face-to-face contacts cannot be transferred to a new region—which represents a cost. We expect that people with stronger local social ties are less likely to move (H2a).

Another local tie that could decrease the likelihood of relocation is homeownership. Unlike renters, owners have to bear substantial transaction costs upon sale of their home (Oswald, 2019). However, our study focuses on a relatively young sample in which only 5% of respondents own a home. The median age at entry into homeownership in Germany is around the mid-thirties (Angelini et al., 2013), while the mean age in our sample is 27.4 years. Given the limited sample size, we refrained from investigating homeownership as an explanation of moving at the start of coresidence.

Regarding working life, partners who are employed are attached to the local labor market. Such local ties to work should reduce the probability of migrating (Mulder & Malmberg, 2014). But how do partners decide if they have a conflict of interest, e.g., if both partners are employed and both have a preference for staying in their place? Contradicting classic microeconomic arguments, individuals might be more interested in their own relative power than in total household benefit (Lundberg & Pollak, 1996). In line with this idea, a person would be less willing to move if relocating downgrades her bargaining position (Abraham et al., 2010). The bargaining position of a person is indicated by her relative resources compared to those of her partner. With respect to moving over longer distances at the start of coresidence, we therefore expect that the partner with more relative resources is less likely to move (H2b). Because we lack data about the second partner’s moving behavior, we cannot test the effect of relative resources on the moving decisions within a couple. However, we can observe across couples whether a person with more relative resources is less likely to move than a person with fewer relative resources.

The basic notion of the bargaining approach leads to gender-neutral expectations, i.e., the relative position has the same effect for men and for women. However, gender norms may also impose costs on couples that do not comply with them (Lundberg & Pollak, 1996). Indeed, the gender role approach emphasizes that women tend to support men’s career- and job-related migration because they are expected to do so—regardless of their own economic position and prospects (Bielby & Bielby, 1992; Cooke, 2008a). In contrast to the relative resources idea, the gender role approach suggests that it is not employment or demographic characteristics that affect a woman’s bargaining position but the perceived social expectation that the female partner should support her male partner and bear the costs of moving. Deviating from such gender norms may lead to sanctions, and in order to avoid these, women might be willing to move regardless of their relative resources. Following from this argumentation, one would expect that women are more likely to move than men at the start of coresidence (H3).

## Previous Research

As noted in the introduction, previous research that explicitly analyses the moving decision at the start of coresidence is scarce. One Swedish study has analyzed the moves of individuals when they establish a joint household with their partner (Brandén & Haandrikman, 2019). It should be noted that, due to data limitations, their sample only included couples who eventually had a first common child or got married; short-lived cohabitations are underrepresented. In line with our Hypothesis 2b, the study showed that a smaller share of relative resources was associated with a higher likelihood of moving at the start of coresidence.

Moreover, in line with Hypothesis 3, the authors found a gendered pattern: the odds of moving at the start of coresidence in Sweden were slightly higher for women, and for long-distance couples (i.e., those who lived more than 50 km apart before they established a joint household), the median distance women moved was 38 km farther than the median distance men moved at the start of coresidence (Brandén & Haandrikman, 2019: p. 445). Other studies on migration around the time of marriage also found indications that women were more likely to move toward men than vice versa (Fan & Huang, 1998; Mulder & Wagner, 1993). There is also evidence that two-gender couples tend to live closer to the man’s than the woman’s parents—likely because the woman moved toward the man (Blaauboer et al., 2011; Løken et al., 2013).

In sum, the existing literature implies that women are more likely to move at the start of coresidence. However, the papers presented here analyze samples of mainly longer-lasting couples, e.g., those who are married or who are on average older than those in our sample. It remains unclear whether these results extend to the migration decision at the start of coresidence among younger couples (of mean age 27 in our empirical analyses), some of whom might have less stable relationships.

## The German Context

In our analyses, we focus on couples living in Germany. With regard to the housing system, Germany stands out for its comparably low homeownership rate of 51.1% in 2019 (as compared with the estimated EU28 average of 69.2%; Eurostat, 2020). Young Germans have weaker incentives to enter homeownership because long-term renting is affordable, secure and accepted (Lennartz & Helbrecht, 2018). While in Great Britain, for example, entry into homeownership is synchronized with union formation, in Germany it is typically postponed until the arrival of a child (Bayrakdar et al., 2019). This is also reflected in the very low share of homeowners in our data of mainly childless young adults in Germany. Although Germany is often rated as gender egalitarian in international comparisons (Aboim, 2010; Voicu, 2017), the combination of a male breadwinner and a female in part-time employment has been the dominant arrangement of families with young children since the mid-1990s (Trappe et al., 2015). Therefore, we might expect that German women are more likely to move in favor of their husbands’ career, while this is less likely to occur in more gender-egalitarian countries, such as Sweden. This idea has been supported in a comparative study of Sweden and Germany (Vidal et al., 2017). The study revealed that in Germany, male-breadwinner and dual-earner couples with a male partner working in a managerial or professional occupation displayed comparatively high migration rates. In Sweden, male-breadwinner families were also more likely to move than other families. But dual-earner couples migrated less often when at least one partner worked in a managerial or professional occupation. The authors interpret this finding as support for the idea that Swedish women are more likely to influence family migration decisions (Vidal et al., 2017).

## Data and Methods

We used 10 waves of the German family panel pairfam, release 10.0 (Brüderl et al., 2019a, b; Huinink et al., 2011). The panel is conducted on an annual basis (data collection between 2008/2009 and 2017/2018) among men and women in three birth cohorts: those who were born in 1991–1993, 1981–1983, or 1971–1973. Within these cohorts, the data collection is based on a 2-stage stratified random sampling procedure. More than 65% of respondents in our sample live in cities with 100,000 inhabitants or more. Data quality tests suggest that pairfam data are highly representative of the corresponding birth cohorts in the general German population (Brüderl et al., 2020). In our sample, we only include couples in which both partners were at least 18 years old. Because our hypothesis 3 focuses on gender differences between partners, we study only two-gender couples. Our units of analysis are 983 individuals that reported that they were living in a LAT partnership in one wave and coresiding with the same partner in the following wave (in an additional analysis we also include couples newly formed between waves—see t﻿he last paragraph of the section “Analytical Strategy”).

In the first wave, the overall response rate was 37%, and the panel stability ranged between 73% and 92% in the following waves (Brüderl et al., 2020). Panel attrition is a phenomenon faced by many longitudinal data collection projects, and this can lead to biased estimates if respondents drop out from the survey in a systematic way. For our study, this is problematic because respondents are more difficult to track after they move (Müller & Castiglioni, 2020) and thus drop out of surveys more often than immobile respondents. If movers systematically differ from stayers, increased dropout rates might distort the results of the analyses of moving behaviors. One way to reduce such bias is the use of weights in the statistical analysis (Trappmann et al., 2015). Therefore, we use the longitudinal weights provided by the pairfam team.^1^

## Outcome Variable: Moving at the Start of Coresidence

Theoretically, we are interested in the variable *moving at the start of coresidence* with regard to both partners. However, in pairfam, information about relocations is collected only for the main respondent, the so-called anchor. Therefore, we focus on the relocation behavior of the anchor at the start of coresidence. In a first step, we identify who has started to coreside with a partner since the last wave. Based on the answers to the questions “In the following, I'll ask you about steady relationships. Do you currently have a partner in this sense?”, “Do you live together with [*name of partner*] in the same dwelling?”, “What is your current marital status?”, and “When did you get married to [*name of partner*] or when was your civil union with [*name of partner*] officially registered?” the pairfam team generated a partnership history for each person. From this, we identified those couples that had an unmarried living-apart-together relationship in wave *t*-1 and reported coresiding with the same partner in wave *t*. All other couples were excluded. In a second step, we used the information about relocation and migration to identify whether a person moved. If a person reported a relocation in the same year as she started coresidence, the outcome variable takes the value 1. If a person entered coresidence but did not report a relocation, the indicator takes the value 0. The majority of anchor persons (64.6% in the full sample) report that they moved at the start of coresidence (Table 1). This seems plausible as this group comprises those who moved in with their partner as well as those who moved to a third place at the start of coresidence.

**Table 1:** Descriptive statistics

	Total sample	Subsample: long-distance couples	Subsample: short-distance couples	*p* value
Distance to partner before move				
Long distance (1 + h)	25.4			
Short distance (< 1 h)	74.6			
Outcome variable: anchor moves				
Yes	64.6	69.8	62.8	
No	35.4	30.2	37.2	0.136
Gender of anchor				
Male	43.2	44.9	42.7	
Female	56.8	55.1	57.3	0.629
Age of anchor person	27.4	27.3	27.4	
Age difference				
Same age	39.3	41.3	38.6	
Anchor older	26.5	24.7	27.0	
Anchor younger	34.2	34.0	34.3	0.818
Educational difference				
Same education	58.1	54.3	59.4	
Anchor higher	21.4	23.2	20.8	
Anchor lower	20.4	22.5	19.8	0.591
Difference in employment status				
Both full-time	34.5	29.4	36.2	
Both other	27.7	27.6	27.8	
Anchor full-time, partner other	16.7	21.4	15.1	
Partner full-time, anchor other	21.1	21.6	20.9	0.271
Children of anchor				
No children	89.1	93.3	87.7	
1 or more children	10.9	6.7	12.3	0.032
Anchor’s travel time to parents				
Long distance (1 + h)	26.0	45.1	19.6	
Short distance (< 1 h)	74.0	54.9	80.5	0.000
Anchor’s housing situation				
Lives with parents	27.8	24.9	28.8	
Own household: crowded	50.7	58.0	48.3	
Own household: uncrowded	21.5	17.1	23.0	0.046
Birth cohort (anchor)				
1991–1993	46.8	47.5	46.6	
1981–1983	39.6	40.2	39.4	
1971–1973	13.5	12.3	14.0	0.875
Region (anchor)				
Western Germany	85.0	85.0	85.1	
Eastern Germany	15.0	15.0	15.0	0.991
Country of birth (anchor)				
Germany	91.1	90.4	91.4	
Other	8.9	9.6	8.6	0.775
Number of partnerships	983	247	736	

Column percentage or means

The *anchor* is the main respondent in the multi-actor design of pairfam. *Short-distance couples* are those who had to travel less than 1 h to meet the partner before coresidence. *Long-distance couples* are those who had to travel 1 h or more. Percentages may not sum to 100 due to rounding. *Source*: pairfam, waves 1–10 (2008/2009–2017/2018). Weighted data (for details of the weighting procedure, see pairfam Group 2020). *p*-values refer to the adjusted Wald test (test of independence with weighted data), comparison of long and short-distance couples

## Explanatory Variables

It seems plausible that relocation decisions are affected by the situation before the actual move. Therefore, in our main analyses we use the measurement of our independent variables in the year before the start of coresidence, i.e., the measurement at time *t*-1. Our first two hypotheses referred to the housing situation before coresidence started. We combine information on housing and living in the parental home into one indicator with three categories: (1) anchor *lives with parents*, (2) living in one’s own crowded home before the move, i.e., one person per room or more—either alone or sharing with others (*own household: crowded*) and (3) living in one’s own household with less than one person per room (*own household: uncrowded*). In pairfam, the question about the number of rooms was posed as follows: “And how many rooms does this apartment (or this house) have?” In case the respondent found this question unclear, the interviewer instructed her or him to count only the rooms that are larger than six square meters, and that are not bathrooms or kitchens. Thus, the number of rooms refers to the number of bedrooms, living rooms and other rooms, such as workrooms. Based on this, we calculated the ratio of individuals living in the household to the number of rooms.

We used three indicators to assess the effect of an individual’s relative resources. We first measure the *age difference* between the partners. Arguably, higher age is associated with a stronger attachment to a location (Fischer & Malmberg, 2001) and to further advancement in the labor market career. Both aspects are associated with a reduced likelihood of moving. The category *same age* includes those partners who are no more than 2 years apart in age, the *anchor older* category includes couples where the anchor is at least 3 years older, and the *anchor younger* category includes couples where the partner is more than 2 years older than the anchor. The second measure of relative resources refers to *educational difference*. We do not have information about partner’s income; thus, we use education as a proxy for earning potential. We classified each partner’s educational attainment based on the International Standard Classification of Education (ISCED 97) and divided it into 3 categories for each partner: *low* (no or lower secondary school degree), *medium* (upper secondary and post-secondary [but non-tertiary]), and *high* (university or college degree). Two partners in the same category were then classified as having the *same education.* Couples in which the anchor had higher education than the partner were categorized as *anchor higher*, those in which the anchor had lower education as *anchor lower*. Our third measure of relative resources is *employment difference*. Here, we assume that employment is an indication of attachment to the local labor market. A person that does not participate in the labor market has no such attachment and therefore a lower cost of relocating than an employed person. By contrast, a person who is employed full-time is well integrated into the local labor market. We assume that a person in full-time employment has a higher income and greater difficulties in finding an adequate job at a new location; thus, the person has a better bargaining position. By contrast, persons who work part-time or are not employed (including unemployment, parental leave, enrolled in education) are assumed to be less integrated into the local labor market and thus their relative resources with respect to employment are smaller. *Both full-time* includes couples with two full-time employed partners. By contrast, the category *both other* refers to couples with two partners who are not employed full-time. Moreover, we distinguish between couples in which the anchor is employed full-time, while the partner belongs to one of the other employment groups (*anchor full-time, partner other*). In the third category are those couples in which the partner is employed full-time, while the anchor belongs to the other category (*partner full-time, anchor other*). It would also be interesting to differentiate between combinations of partners who are employed part-time, inactive, unemployed, or enrolled in education which we now group in the *other* category. However, with more categories, the number of persons in some of the categories were quite small.

We used two measures of local ties. The first is whether the anchor has any *children*. The literature shows that moving can have negative effects on children’s well-being (Coley & Kull, 2016). Because parents may anticipate these negative effects, they are likely to have a preference to stay in their home (or at least in the neighborhood) when forming a joint household. Our second measure of local ties is *anchor’s travel time to the closest parent*. Parents are a central source (and/or recipient) of social support. Small distances facilitate interaction; thus, parents that live in geographic proximity are associated with a person’s immobility (Ermisch & Mulder, 2019; Hünteler & Mulder, 2020). We distinguish between those who have to travel 1 h or more to meet their closest parent from those who have to travel less than 1 h. If a respondent’s parents had passed away before the interview this person was categorized into the long-distance group.

As control variables, we included the anchor’s gender (in the models that are not separated by gender) and age at time of interview (in years). In order to account for potential changes over cohorts, we controlled for birth cohort, i.e., those born in the years (1) 1991–1993, (2) 1981–1983 or (3) 1971–1973. In Eastern Germany, family behaviors, especially marriage behaviors, generally differ from those in Western Germany (Kreyenfeld et al., 2016), therefore we control for the region a person lives in at the time of interview. Moreover, international immigrants might hold more traditional gender role attitudes. Thus, we control whether a person was born outside of Germany, which was the case for 9% of respondents in the total sample.

## Analytical Strategy

The aim of this study is to identify factors that are associated with whether a person moves or has the partner move in at the start of coresidence. Theoretically, we expect that the mechanism underlying the moving decision differs between long- and short-distance moves. Therefore, we estimate separate models by distance. Unfortunately, the distance moved by the partners is not reported in pairfam. As a proxy, we rely on the information whether a couple has been living in a short-distance relationship (less than 1 h travel distance) or long-distance relationship (1 h or more travel distance) before coresidence started. We assume that a mover who had a long-distance relationship is more likely to move over a larger distance when starting coresidence.^2^ The vast majority of couples had a short-distance relationship (*n* = 736; 75%), while 25% (*n* = 247) had a long-distance relationship before the start of coresidence.

In a first step, we discuss the descriptive statistics of the full sample, as well as the samples of long-distance and short-distance couples. Moreover, in additional analyses, we show the descriptive statistics by distance and gender (Table 3 in Appendix). In a second step, we estimate multiple logistic regressions to identify factors that are associated with moving versus having the partner move in. Again, we analyze short- and long-distance couples separately. In order to explore whether relative resources play out differently for men and women, we perform separate regressions for men and women (see Appendix).

One limitation of our study might be selectivity with regard to the speed of union formation. Those who were not in a relationship in wave *t*-1 but entered coresidence between two interviews were excluded from our main sample for lack of information about partner characteristics and distance in wave *t*-1. In an additional analysis, we re-estimated our regression analyses including those couples who started both their relationship and coresidence since the last wave. This increased our sample size by 31 observations. The results of these additional analyses were very similar to the results we present and are therefore not shown. In other words, we did not find any indication that the duration of a LAT relationship affects the associations between the independent variables and the probability of moving at the start of coresidence.

## Descriptive Results

Table 1 shows the descriptive statistics for the total sample as well as short- and long-term couples separately. We have also tested whether differences between short- and long-distance couples are statistically significant (see *p*-values in Table 1). The respondents in our sample are relatively young with a mean age of 27.4. In long-distance couples (69.8%), anchors move more often than in short-distance couples (62.8%). However, this difference is statistically insignificant. We find a significant difference between long- and short-distance couples for the variable *anchor’s travel time to parents*. 54.9% of anchors in long-distance relationships live in close proximity to their parents (i.e., they have to travel less than 1 h to meet with their closest parent). Among those in short-distance relationships, the share that live close to their parents is considerably higher, at 80.5%. One potential explanation for this finding might be that persons in long-distance relationships are generally more mobile and thus live farther away from their family than persons in short-distance relationships. Moreover, there is a marginally significant difference between long- and short-distance couples with regard to anchor’s housing situation (*p* = 0.089). In long-distance couples it is more common that the anchor lives in a crowded home than in short-distance couples. The other covariates do not differ significantly between short- and long-distance couples.

In Table 3 (see Appendix), we present additional descriptive analyses for men and women separately. Our results show that women have on average lower levels of relative resources than men. These differences are statistically significant.

## Multiple Regression Results

In our main analyses, we estimated three logistic regression models of the anchor respondent’s moving behavior at the start of coresidence. Model 1 in Table 2 presents the results for the full sample. Model 2 presents the regression results for the smaller sample of couples who were in a long-distance relationship before forming a joint household. Model 3 refers to couples in short-distance relationships before the move. In order to explore potential gender differences in the role of relative resources and local ties, we estimate separate regressions by gender. The results of these analyses are presented in Tables 4 and 5 in the Appendix.

**Table 2:** Logistic regression analysis, outcome: anchor moves at the start of coresidence

	Model 1 Total sample		Model 2 Subsample: long-distance couples		Model 3 Subsample: short-distance couples	
	AME	*p* value	AME	*p* value	AME	*p* value
Distance to partner before move						
Long distance (1 + h)	0.036	0.395				
Short distance (< 1 h)	0					
Gender of anchor						
Male	0		0		0	
Female	− 0.070	0.134	− 0.047	0.567	− 0.068	0.216
Age of anchor person	− 0.006	0.496	− 0.001	0.972	− 0.005	0.635
Age difference						
Same age	0		0		0	
Anchor older	− 0.040	0.443	0.049	0.622	− 0.075	0.219
Anchor younger	0.073	0.107	0.103	0.171	0.072	0.176
Educational difference						
Same education	0		0		0	
Anchor higher	− 0.078	0.154	− 0.267	0.004	− 0.023	0.716
Anchor lower	0.029	0.566	0.056	0.563	0.016	0.784
Difference in employment status						
Both full-time	0		0		0	
Both other	− 0.075	0.179	− 0.095	0.422	− 0.059	0.374
Anchor full-time, partner other	− 0.109	0.056	− 0.116	0.235	− 0.118	0.096
Partner full-time, anchor other	− 0.066	0.267	0.032	0.782	− 0.104	0.136
Children of anchor						
No children	0		0		0	
1 or more children	− 0.085	0.279	− 0.137	0.302	− 0.076	0.403
Anchor’s travel time to parent(s)						
Long distance (1 + h)	0		0		0	
Short distance (< 1 h)	− 0.093	0.118	− 0.115	0.221	− 0.099	0.141
Anchor’s housing situation						
Lives with parents	0.216	0.001	− 0.033	0.717	0.282	0.000
Own household: crowded	0.160	0.004	− 0.081	0.282	0.225	0.000
Own household: uncrowded	0		0		0	
Birth cohort (anchor)						
1991–1993	0		0		0	
1981–1983	− 0.017	0.827	− 0.121	0.363	0.010	0.912
1971–1973	0.021	0.896	− 0.190	0.567	0.045	0.816
Region (anchor)						
Western Germany	0		0		0	
Eastern Germany	0.062	0.176	0.046	0.531	0.070	0.197
Country of birth (anchor)						
Germany	0		0		0	
Other	− 0.143	0.086	− 0.058	0.667	− 0.181	0.067
Number of partnerships	983		247		736	

Average marginal effects

The *anchor* is the main respondent in the multi-actor design of pairfam. Short-distance couples are those who had to travel less than 1 h to meet the partner before coresidence. Long-distance couples are those who had to travel 1 h or more. *Source*: pairfam, waves 1–10 (2008/2009–2017/2018). Weighted data (for details of the weighting procedure, see pairfam Group 2020)

We present average marginal effects (AME) and *p*-values. The average marginal effect is the mean of the marginal effects for each combination of covariates in the dataset. It represents the average change in the probability of seeing a specific outcome when we alter the respective independent variable from the reference to a different category based on our sample. In our models, the AME of a covariate can be interpreted as the change in the anchor person’s probability of moving at the start of coresidence for the value of a variable compared to the reference category of this variable. While conclusions based on a direct comparison of odds ratios across different models can be erroneous, comparisons of AME are unproblematic (Mood, 2010). Because some of our sub-samples are small, we also discuss results with *p*-values between 0.05 and 0.10.

In Model 1 in Table 2, the anchor’s employment status, her/his housing situation and country of birth are significantly associated with the anchor’s likelihood of relocation. For the partners’ employment status, our results indicate that if an anchor person is employed full-time, while the partner’s status is *other*, the anchor is less likely to move (AME = − 0.109, *p* = 0.056) than in cases where both partners are employed full-time (reference). With regard to the housing situation, we find that those who live with their parents have a 21.6% higher probability of moving (*p* = 0.001) compared to those who live in their own, uncrowded home (reference). Those living in their own crowded home also have an increased probability of moving (AME = 0.161, *p* = 0.004). Moreover, persons who are born outside Germany are less likely to move at the start of coresidence (AME = − 0.143, *p* = 0.086) than those born in Germany (reference). In Model 1, we also controlled for the distance between partners before the move. However, the effect is small and insignificant.

Model 2 presents the results for the sample of long-distance couples. Only one variant of the relative resources is statistically significant in this model: the educational difference between partners. In couples where the anchor has higher education than the partner, the anchor is less likely to move (AME = − 0.267, *p* = 0.004) than in couples with the same educational level (reference). We do not find evidence for our hypothesis 2a that social ties are associated with a lower probability of moving: the coefficients of the variables *Children of anchor* and *Anchor’s travel time to parent(s)* are small with large *p*-values. The other coefficients in this model are also rather small and statistically insignificant.

With regard to short-distance couples (Model 3), they seem to drive the results found in Model 1. Theoretically, for short-distance moves we expected that a person’s housing situation would be especially important in making relocation decisions. In line with Hypotheses 1a and 1b, persons who live in their parents’ home and those who live in their own crowded home are more likely to move than those who live in their own uncrowded home. Moreover, we find that an employment situation with an anchor employed full-time and a partner with *other* employment status is associated with a lower probability of moving (AME = − 0.118, *p* = 0.096) compared to couples where both partners are employed full-time (reference). Theoretically, we did not expect such differences by relative resources in short-distance couples. With regard to the other control variables, we found that anchors born outside Germany are less likely to move than German-born persons. This might indicate that local social networks are more relevant for immigrants than for natives.

In our third hypothesis, we expected that women are more likely to move than men at the start of coresidence. We do not find evidence for this in Models 1, 2 and 3. Also in a bivariate analysis (result not shown), gender was not significantly associated with the probability of moving. In order to explore how relative resources (and other couple characteristics) affect men and women differently, we performed additional analyses by gender. Table 4 in the Appendix shows two interesting gender differences. First, women who are younger than their partner have an increased probability of moving (AME = 0.093, *p* = 0.076) relative to those who are the same age as their partners (reference). This effect is not found among men. Second, women who live in close proximity to at least one of their parents are less likely to move (AME = − 0.151, *p* = 0.006) than those who do not live close to their parents (reference). This pattern persists for women in long- and short-distance couples (Table 5 in Appendix), while it does not appear for men. The analyses by gender and distance also reveal additional differences between subgroups. For example, women from the early 1970s and early 1980s birth cohorts were significantly less likely to move than those from the early 1990s cohort. However, it should be noted that the case numbers in the smaller sample of long-distance relationships are quite small in some of the categories, e.g., *n* = 28 women belong to the 1970s cohort and *n* = 24 women lived in their own uncrowded home. Therefore, we refrain from drawing strong conclusions about these gender differences.

## Discussion

In this study, we analyzed couples’ decisions about who moves and who stays in their home at the start of coresidence. Our findings showed differences in the conditions associated with relocation depending on partners’ travel distance before the formation of a joint household. For short-distance couples, the housing situation seemed to be relevant for the moving decision. Persons who live with their parents as well as those who have their own crowded home were more likely to move than those who live in their own uncrowded home. This supports our hypotheses 1a and 1b and indicates that household formation in the context of a short-distance move is a practical reaction to new space demands, as has been suggested for family moves, e.g., in the period around the birth of a child (Rossi, 1955). Moreover, our findings add to the emerging literature on the relevance of the housing situation for partnership development and moving decisions (Coulter & Thomas, 2019; Krapf & Wagner, 2020; van Damme et al., 2018). Moreover, we found that relative resources were relevant for the decision to relocate in short-distance couples as well. Respondents who were employed full-time and had a partner who was in the *other* employment category were less likely to move than respondents in a couple with two partners employed full-time.

In long-distance couples, our findings also partly support the hypotheses that relative resources (and family ties for women) matter in determining who moves at the start of coresidence. Respondents with higher educational attainment than their partner are less likely to move than those with the same educational level. This was the case for both men and women. For women, we found that those who are younger than their partner had an increased probability of moving than those with the same age. By contrast, we did not find evidence for an effect of relative resources with regard to employment status. This pattern might indicate the central role of partners’ earning potential (that is associated with their education) in the decision to move over a long distance.

We did not find a strong main effect of gender on the probability of moving at the start of coresidence. However, in separate analyses we found that the factors that are associated with moving seemed to differ between men and women. Women in long- and short-distance relationships who live in close proximity to at least one of their parents had a lower probability of moving than those who live further away from their parents. For men, this effect was not found. One reason for this might be that daughters provide care for their parents more frequently than sons (Leopold et al., 2014). Providing support to parents (or anticipating the need to provide care in the future) might keep women from moving away from the place where their parents live.

Prior research finds that women are more likely to move compared to men at the start of coresidence in Sweden (Brandén & Haandrikman, 2019) and in other studies about gendered moving patterns within families (Fan & Huang, 1998) (although differences were not very large). Moreover, in Germany family migration seems to be driven more by the man’s employment and position than the woman’s (Vidal et al., 2017). We therefore expected that moving at the start of coresidence would also be more likely among women than men in Germany. However, in our data, we do not find a strong gender difference in the probability to move. Women who live close to their parents were even less likely to move—which contradicts the gender hypothesis. These findings might be associated with our focus on partnership behaviors among young individuals, with a mean age of 27 years in our sample. Brandén and Haandrikman (2019) analyzed a sample consisting exclusively of couples that later married or had a child. Gendered attitudes and behaviors often become more traditional after a child is born (Baxter et al., 2015; Endendijk et al., 2018). This might also extend to the moving decision. Indeed, it has been shown that gendered patterns in family migration occur for mothers, but not for childless women (Cooke, 2001). Therefore, our findings might be evidence of egalitarian moving behaviors among young couples. Apart from the insignificant main effect of gender, we did find differences associated with relative resources for men and women in short-distance relationships—which we actually expected to find for persons in long-distance relationships. However, we acknowledge that our findings have to be interpreted with caution because the sample sizes in some categories were quite small.

Given the few existing studies on the topic, we need more research about moving at the start of coresidence. First of all, we need larger datasets to enlarge statistical power when assessing moving decisions in sub-categories, i.e., for long- versus short-distance moves and among men versus women. Moreover, we only had information about whether the respondent moved or not but we were unable to distinguish between those couples in which the respondent moved in with the partner or both moved to a new home. This limitation in the measurement of our outcome variable might lead to a biased estimation of the role of relative resources in the moving decision. To illustrate this point, we compare two hypothetical couples, one with similar relative resources, and one where the partner has more resources than the anchor. Following from the bargaining approach, partners with similar relative resources would agree to move to a new place as a reasonable compromise. By contrast, in the couple where the partner has more resources than the anchor, the anchor moves to the partner’s place. With our data, we group both cases together into the “anchor moves”-category, which might obscure part of the measured effect of relative resources. Another data limitation is related to the measurement of distance moved. This information is not collected for the partner in the pairfam survey. Instead, we used the distance between the partners before they established a joint household. If the couple was in a long-distance relationship, it is possible that both partners moved to a place that is geographically between their two previous residences and potentially one or both partners would move a short distance. In this example, our procedure leads to measurement error. In order to avoid such measurement problems, in future surveys more data from LAT partners should be collected.

## Appendix

See Tables 3, 4 and 5.

**Table 3:** Descriptive statistics by gender and *p*-values

	Long-distance couples			Short-distance couples		
	Men	Women	*p* value	Men	Women	*p* value
Outcome variable: anchor moves						
Yes	65.5	73.3		63.1	62.8	
No	34.5	26.7	0.287	36.9	37.4	0.924
Age of anchor person	28.9	26.0	0.002	28.9	26.4	0.002
Age difference						
Same age	32.1	48.8		39.4	38.1	
Anchor older	48.8	5.1		48.3	11.2	
Anchor younger	19.1	46.1	0.000	12.4	50.7	0.000
Educational difference						
Same education	58.9	50.6		59.2	59.6	
Anchor higher	27.7	19.6		27.8	15.7	
Anchor lower	13.5	29.8	0.059	13.1	24.7	0.015
Difference in employment status						
Both full-time	31.2	28.0		38.7	34.4	
Both other	24.4	30.1		28.2	27.5	
Anchor full-time, partner other	34.1	11.0		27.0	6.3	
Partner full-time, anchor other	10.3	30.9	0.001	6.2	31.9	0.000
Children of anchor						
No children	96.3	90.8		86.6	88.6	
1 or more children	3.7	9.2	0.073	13.4	11.4	0.573
Anchor’s travel time to parents						
Long distance (1 + h)	32.7	55.2		18.8	20.1	
Short distance (< 1 h)	67.3	44.8	0.007	81.2	80.0	0.758
Anchor’s housing situation						
Lives with parents	25.6	24.3		32.1	26.3	
Own household: crowded	53.1	62.0		43.5	51.8	
Own household: uncrowded	21.2	13.7	0.359	24.4	21.9	0.086
Birth cohort (anchor)						
1991–1993	32.9	59.4		36.2	54.4	
1981–1983	50.1	32.2		45.4	35.0	
1971–1973	17.1	8.4	0.002	18.4	10.6	0.002
Region (anchor)						
Western Germany	86.7	83.6		86.3	84.1	
Eastern Germany	13.3	16.4	0.547	13.7	15.9	0.481
Country of birth (anchor)						
Germany	87.8	92.5		93.7	89.7	
Other	12.2	7.5	0.320	6.4	10.3	0.416
Number of partnerships	111	136		314	422	

Column percentage or means

The *anchor* is the main respondent within the multi-actor design of pairfam. *Short-distance couples* are those who had to travel less than 1 h to meet the partner before coresidence. *Long-distance couples* are those who had to travel 1 h or more. Percentages may not sum to 100 due to rounding. *Source*: pairfam, waves 1–10 (2008/2009–2017/2018). Weighted data (for details of the weighting procedure, see pairfam Group 2020). *p*-values refer to the adjusted Wald test (test of independence with weighted data), comparison of men and women

**Table 4:** Logistic regression analysis by gender, outcome: anchor moves at the start of coresidence

	All couples			
	Men		Women	
	AME	*p* value	AME	*p* value
Distance to partner before move				
Long distance (1 + h)	0.015	0.807	0.031	0.583
Short distance (< 1 h)	0		0	
Age of anchor person	− 0.012	0.412	0.003	0.784
Age difference				
Same age	0		0	
Anchor older	− 0.045	0.482	− 0.153	0.141
Anchor younger	− 0.045	0.605	0.093	0.076
Educational difference				
Same education	0		0	
Anchor higher	− 0.109	0.127	− 0.016	0.843
Anchor lower	0.015	0.863	0.033	0.547
Difference in employment status				
Both full-time	0		0	
Both other	0.032	0.691	− 0.136	0.057
Anchor full-time, partner other	− 0.127	0.098	− 0.049	0.557
Partner full-time, anchor other	− 0.052	0.661	− 0.098	0.126
Children of anchor				
No children	0		0	
1 or more children	− 0.145	0.240	0.025	0.724
Anchor’s travel time to parents				
Long distance (1 + h)	0		0	
Short distance (< 1 h)	− 0.087	0.243	− 0.151	0.006
Anchor’s housing situation				
Lives with parents	0.162	0.074	0.263	0.001
Own household: crowded	0.161	0.047	0.156	0.032
Own household: uncrowded	0		0	
Birth cohort (anchor)				
1991–1993	0		0	
1981–1983	0.144	0.161	− 0.189	0.055
1971–1973	0.185	0.398	− 0.198	0.379
Region (anchor)				
Western Germany	0		0	
Eastern Germany	0.091	0.185	0.043	0.476
Country of birth (anchor)				
Germany	0		0	
Other	− 0.160	0.211	− 0.087	0.346
Number of partnerships	425		558	

Average marginal effects

The *anchor* is the main respondent in the multi-actor design of pairfam. Short-distance couples are those who had to travel less than 1 h to meet the partner before coresidence. Long-distance couples are those who had to travel 1 h or more. *Source*: pairfam, waves 1–10 (2008/2009–2017/2018). Weighted data (for details of the weighting procedure, see pairfam Group 2020)

**Table 5:** Logistic regression analysis by distance and gender, outcome: anchor moves at the start of coresidence

	Long-distance couples				Short-distance couples			
	Men		Women		Men		Women	
	AME	*p* value	AME	*p* value	AME	*p* value	AME	*p* value
Age of anchor	− 0.032	0.169	0.027	0.286	− 0.007	0.641	0.005	0.715
Age difference								
Same age	0		0		0		0	
Anchor older	0.106	0.285	− 0.013	0.941	− 0.111	0.122	− 0.161	0.164
Anchor younger	0.108	0.447	0.111	0.172	− 0.089	0.372	0.099	0.112
Educational difference								
Same education	0		0		0		0	
Anchor higher	− 0.272	0.017	− 0.281	0.063	− 0.028	0.726	0.042	0.630
Anchor lower	0.170	0.194	− 0.058	0.590	− 0.037	0.710	0.045	0.477
Difference in employment status								
Both full-time	0		0		0		0	
Both other	− 0.151	0.283	− 0.013	0.941	0.114	0.203	− 0.154	0.061
Anchor full-time, partner other	− 0.199	0.084	0.004	0.984	− 0.129	0.160	− 0.065	0.552
Partner full-time, anchor other	0.118	0.386	0.087	0.601	− 0.167	0.217	− 0.133	0.068
Children of anchor								
No children	0		0		0		0	
1 or more children	0.015	0.934	− 0.135	0.518	− 0.161	0.224	0.046	0.559
Anchor’s travel time to parent(s)								
Long distance (1 + h)	0		0		0		0	
Short distance (< 1 h)	− 0.114	0.178	− 0.180	0.084	− 0.100	0.315	− 0.145	0.031
Anchor’s housing situation								
Lives with parents	0.025	0.853	− 0.045	0.698	0.225	0.043	0.328	0.000
Own household: crowded	0.088	0.412	− 0.193	0.036	0.187	0.055	0.239	0.004
Own household: uncrowded	0		0		0		0	
Birth cohort (anchor)								
1991–1993	0		0		0		0	
1981–1983	0.128	0.443	− 0.403	0.003	0.194	0.066	− 0.193	0.096
1971–1973	0.350	0.165	− 0.656	0.004	0.181	0.467	− 0.196	0.469
Region (anchor)								
Western Germany	0		0		0		0	
Eastern Germany	0.001	0.991	0.132	0.131	0.128	0.109	0.036	0.606
Country of birth (anchor)								
Germany	0		0		0		0	
Other	− 0.261	0.088	0.150	0.087	− 0.067	0.598	− 0.186	0.104
Number of partnerships	112		135		313		423	

Average marginal effects

The *anchor* is the main respondent in the multi-actor design of pairfam. Short-distance couples are those who had to travel less than 1 h to meet the partner before coresidence. Long-distance couples are those who had to travel 1 h or more. *Source*: pairfam, waves 1–10 (2008/2009–2017/2018). Weighted data (for details of the weighting procedure, see pairfam Group 2020)

## Footnotes

1 We followed the procedure recommended by the pairfam team; see the do-file published by the pairfam Group (2020).

2 In case that an anchor person, who reported a long-distance relationship in the last wave and had the partner move in at the start of coresidence, we can conclude that the partner moved over a long distance. However, it should be noted that in case that the anchor moved, it is possible that both partners move to a place that is geographically in between their two previous residences and thus one partner or both partners would fall into the “short-distance move” category. In such a case, our procedure leads to measurement error.
